# Preoperative diagnosis of knee cartilage, meniscal, and ligament injuries by magnetic resonance imaging

**DOI:** 10.1186/s40634-023-00595-y

**Published:** 2023-04-20

**Authors:** Yasuaki Nakagawa, Shogo Mukai, Sayako Sakai, Ryota Nakamura, Motoi Takahashi, Shinnosuke Nakagawa

**Affiliations:** 1 Clinical Research Center, National Hospital Organization Kyoto Medical Center, 1-1 Fukakusa Mukaihata-Cho, Fushimi-Ku, Kyoto, 612-8555 Japan; 2 Department of Orthopaedic Surgery, Japan Baptist Medical Foundation, Kyoto, Japan; 3 Department of Orthopaedic Surgery, National Hospital Organization Kyoto Medical Center, Kyoto, Japan; 4 National Hospital Organization Kyoto Medical Center, Kyoto, Japan

**Keywords:** Knee articular cartilage, Magnetic resonance imaging, Diagnosis, Meniscus, Anterior cruciate ligament

## Abstract

**Purpose:**

The purpose of the study was to report on the current accuracy measures specific to 1.5-Tesla MRI of the knee in the patient population prone to injuries of the anterior cruciate ligament (ACL), the menisci, and the articular cartilage.

**Methods:**

We accrued patients between January 2018 through August 2021 who underwent a preoperative MRI and were diagnosed with an articular cartilage injury either due to unevenness of articular cartilage in T2-weighted sequences or due to the irregularity of subchondral bone in T1-weighted sequences. All patients were treated arthroscopically. Sensitivity, specificity, and accuracy were calculated for the detection of ACL, meniscus, and cartilage injuries. A *P*-value of < 0.05 represented statistical significance.

**Results:**

One-hundred and forty-seven cases which included 150 knee joints were enrolled in this study. The mean age at the time of surgery was 42.9 years-old. The sensitivity in the diagnosis of ACL injuries was significantly greater than that in the diagnosis of cartilage injuries (*P* = 0.0083). The ratios of the equality of operative indication in 6 recipient sites were found to be between 90.0% and 96.0%. The diagnostic critical point was within a 1 cm in diameter.

**Conclusion:**

The diagnostic sensitivity in cartilage injuries was significantly lower than ones of ACL and meniscal injuries. The ratios of the equality of operative indication was determined to be between 90.0% and 96.0%, if we consider the unevenness of articular cartilage or the irregularity of subchondral bone.

**Level of evidence:**

Level III, Prospective diagnostic cohort study.

**Supplementary Information:**

The online version contains supplementary material available at 10.1186/s40634-023-00595-y.

## Introduction

Osteoarthritis (OA) in the knee is a progressive joint disease characterized by knee pain, disability and articular cartilage loss. Identifying structural features that precede clinically evident disease is critical for the implementation of early interventions to slow the disease trajectory [5]. Currently, plain radiography remains the gold standard for morphological assessment of OA, and radiographic joint space narrowing is used as the criterion for developing disease-modifying drugs. Joint space narrowing is only a crude marker for cartilage thinning and lacks significant sensitivity [15]. Magnetic resonance imaging (MRI), by contrast, enables direct view of cartilage volume. More importantly, MRI provides detailed information about early soft tissue structural changes which are not detectable on radiographs but are crucial for assessment of disease, monitoring the progression, and for treatment planning [8, 14].

Arthroscopy is considered the gold-standard for the diagnosis of internal knee pathologies in addition to being a minimally invasive surgical procedure for the treatment of intra-articular lesions [11]. Yet, as in all surgical procedures, it should be recommended judiciously for appropriate indications. Recently, MRI has become more widely used in the evaluation of intra-articular knee lesions [26], though initial studies were completed nearly 30 years ago [13]. The accuracy of MRI for knee articular cartilage lesions has become more controversial several contemporary studies reporting sensitivity as low as 15% and as high as 60% depending on the characteristics and locations of the lesions [25, 28, 34].

**Fig. 1:**
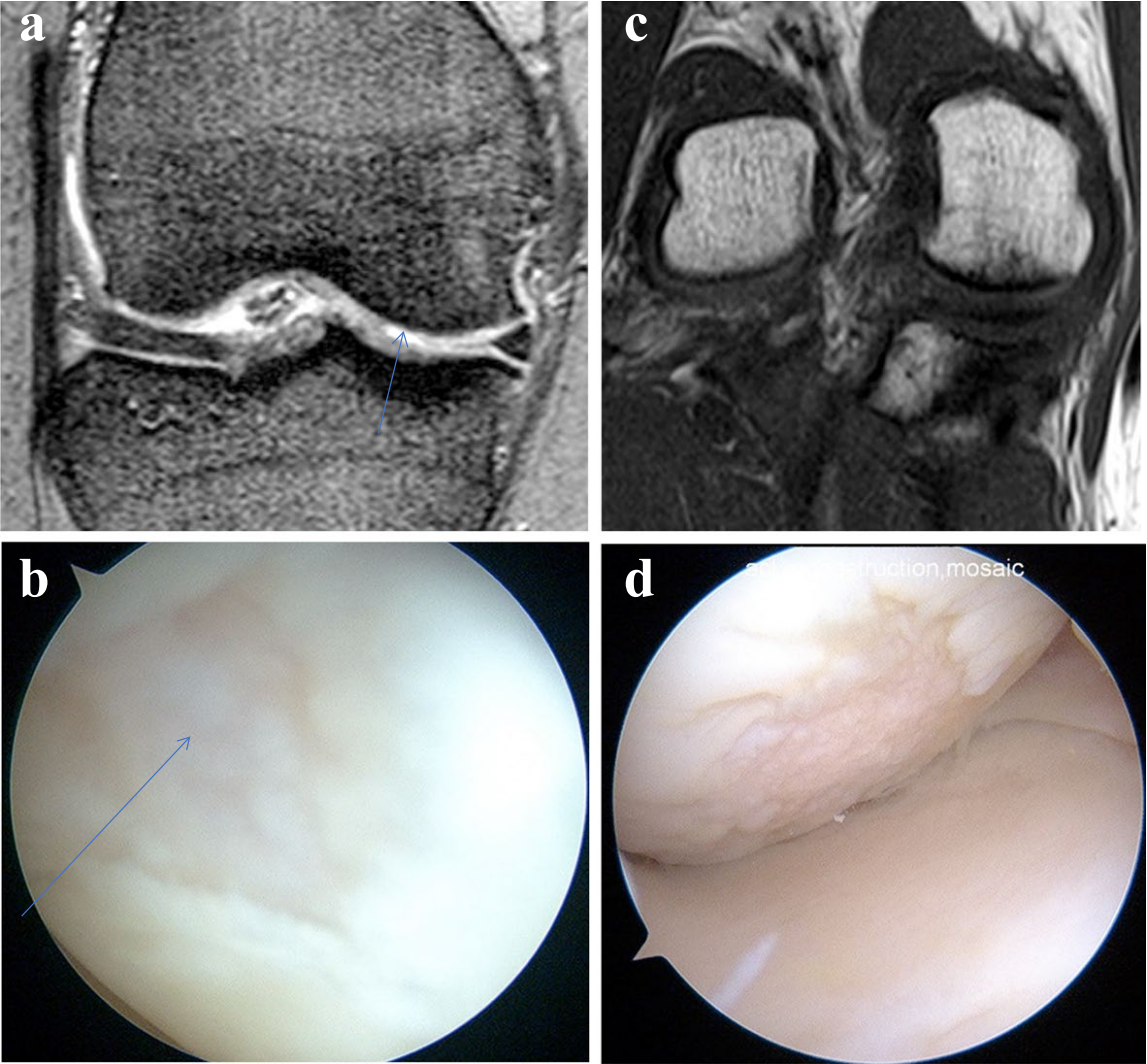
**a** The unevenness of articular cartilage with effusions in T2-weighted sequences in the right medial femoral condyle (arrow). **b** The arthroscopic view. The arrow showed cartilage injury in MFC. **c** The irregularity of subchondral bone in T1-weighted sequences in the right medial femoral condyle. **d** The arthroscopic view

MRI findings consistent with articular cartilage injuries include the unevenness of articular cartilage with inflammatory effusion in T2-weighted sequences or the irregularity of subchondral bone in T1-weighted sequences (Fig. 1). T2-weighted images provide greater contrast between cartilage surfaces and effusions and can detect subtle changes such as fibrillation [17]. The purpose of the current study was to further identify and define measurements specific to 1.5-Tesla MRI of the knee in the patient population presenting with injuries likely to involve the anterior cruciate ligament (ACL), the menisci, and the articular cartilage. We hypothesized that MRI accuracy would lie between 80 to 90% for the menisci and ACL and would lie between 60 to 70% for the articular cartilage. We also hypothesized that the diagnostic critical point of cartilage injuries was at least 1 cm in diameter. This is a clinical study by the orthopaedic surgeons who were specialists of knee surgeries.

## Materials and methods

We enrolled all patients who underwent a diagnostic preoperative MRI followed by arthroscopy of the knee between January 2018 and August 2021 in this study. Inclusion criteria were as follows: (1) patient age older than 9 year, (2) primary knee arthroscopy, (3) MRI performed at the same institution, (4) time interval between the MRI scan and arthroscopy shorter than 3 months. Exclusion criteria were as follows: (1) revision arthroscopy, (2) arthroscopic-assisted fracture reduction surgery, and (3) multi-ligament knee surgery. The status of the ACL, the menisci, and the articular cartilage was collected from the surgical reports and from their respective preoperative MRI reports. Preoperative MRI reports were written by the knee specialist orthopaedic surgeon who examined his patients. All procedures were reviewed and approved by the research ethics committee of our hospital (KMC 13–20).

All MRI images were obtained on a clinical 1.5 T unit (Achieva, Royal Philips, Amsterdam, the Netherlands) using a standard receive-only 8-channel SENSE knee coil. The MRI used a 1.5-Tesla magnet and the standard protocol included the Turbo Spin Echo technique including proton density, T2- and T1-weighted sequences with fat suppression and Short Tau Inversion Recovery sequences with sagittal, coronal, and axial cuts. The MRI criteria used to define pathological lesions of the menisci and ACL were included the following. An intra-meniscal signal extending to an articular surface possibly including distortion of the normal meniscal shape representing a clinically meaningful tear [7], discontinuity of the ACL fibers, wavy appearance, and an angle of less than 45 degree between the distal ACL fibers and the tibia [26] representing a complete tear of ACL; one bruise at the anterior or central lateral femoral condyle and posterolateral tibial plateau with or without countercoup bone contusion at the posteromedial tibial plateau [26] are also secondary signs of an ACL tear.

The MRI criteria used to diagnose articular cartilage injuries included unevenness of articular cartilage with associated effusions in T2-weighted sequences and the irregularity of subchondral bone in T1-weighted sequences (Fig. 1). When evaluating the preoperative MRI, we diagnosed the articular cartilage injury grades in the classification of International Cartilage Repair Society (ICRS) [2], and the recipient area using a ruler in MRI. Six recipient sites were assessed in the following: medial femoral condyle (MFC), lateral femoral condyle (LFC), medial tibia (MT), lateral tibia (LT), trochlea (TR), and patella (P). We compared the preoperative MRI data with the arthroscopic data. We also calculated the simultaneous injuries ratio of cartilage in MFC and medial meniscus, and cartilage in LFC and lateral meniscus.

In ACL, meniscus and cartilage injuries, whether or not they were normal or abnormal, sensitivity {(true positive)/(true positive) + (false negative)}, specificity {(true negative)/(true negative) + (false negative)}, and accuracy {(true positive) + (true negative)/(total)} were calculated. In cartilage injuries, the ratio of diagnosing the same grade in ICRS classification was also calculated. Because the recipient sites with grade 3 or 4 of ICRS required cartilage reconstruction surgery such as autologous chondrocyte implantation or autologous osteochondral grafts [16, 27], we also measured the operative indication ratio which is defined as the diagnostic ratio between less than grade 2 and more than grade 3.

The Mann–Whitney U-test and the chi-square test were used to perform statistical analyses of the various scores. The level of statistical significance was set to a *P* value of < 0.05.

## Results

One-hundred and forty-seven cases including 150 knee joints were enrolled in this study. There were 68 men and 79 women, 70 right knee joints and 80 left ones. The mean operative age was 42.9 years-old (range 9 to 81 years). Diagnosed diseases in this study are presented in Table 1. Normal cases existed 113 knees in medial meniscus, 91 knees in lateral knees and 105 knees in anterior cruciate ligament. The rates of ICRS classification in 6 recipient sites using arthroscopy are presented in Table 2. The most frequent site of abnormal findings in cartilage was the MFC. The most frequent site of injuries rated higher than a grade 3 was also the MFC, and the second site was the TR. The injuries in the medial meniscus were detected in 37 cases (24.7%), and the lateral meniscus injuries were detected in 59 cases (39.3%). The ratio of the cartilage injuries in the LFC was 18.6% in all of the cases of injuries in the lateral meniscus, and the ratio of the cartilage injuries in MFC was 59.5% in all cases of injuries of the medial meniscus.

**Table 1:** Diagnosed diseases

Basic diseases	Case number
Ligament injury	42 cases
Meniscus injury	33 cases
Cartilage injury	24 cases
Osteoarthritis	19 cases
Osteonecrosis	15 cases
Knee synovitis	8 cases
Osteochondritis Dissecans	7 cases
Osgood disease	2 cases

**Table 2:** The rates of ICRS classification in 6 recipient sites using arthroscopy

ICRS grade	Grade 0	Grade 1	Grade 2	Grade 3	Grade 4
MFC	52.4%	0%	23.1%	11.6%	12.9%
LFC	84.7%	0%	2.7%	4.7%	8.0%
MT	76.5%	1.3%	15.4%	1.3%	5.4%
LT	79.3%	0%	12.7%	2.0%	6.0%
TR	66.0%	0%	15.3%	8.0%	10.7%
P	72.7%	3.3%	16.7%	1.3%	6.0%

*MFC* Medial femoral condyle

*LFC* Lateral femoral condyle

*MT* Medial tibial plateau

*LT* Lateral tibial plateau

*TR* Trochlea

*P* Patella

The sensitivity, specificity, and accuracy of intra-articular structures are presented in Table 3, and those of the 6 recipient sites in articular cartilage are presented in Table 4. The sensitivity in ACL injuries was significantly higher than that in cartilage injuries (*P* = 0.0083), and the sensitivity in injuries of the medial meniscus (MM) was significantly higher than those in the cartilage injuries of MT (*P* = 0.0465) and P (*P* = 0.0233). In cartilage injuries, the sensitivity in MFC (*P* = 0.0697) and LT (*P* = 0.0640) were both higher than that in P. There were no significant differences in specificity and accuracy.

**Table 3:** Sensitivity, specificity, and accuracy of intraarticular structures

	Cartilage total	Medial meniscus	Lateral meniscus	Anterior cruciate ligament
Sensitivity	0.482	0.838	0.678	0.933
Specificity	0.989	0.841	0.868	0.981
Accuracy	0.802	0.840	0.793	0.967

**Table 4:** Sensitivity, specificity, and accuracy of the site in articular cartilage

	MFC	LFC	MT	LT	TR	P
Sensitivity	0.657	0.391	0.343	0.355	0.392	0.317
Specificity	1.0	0.992	1.0	0.992	0.980	0.972
Accuracy	0.905	0.90	0.846	0.860	0.780	0.793

*MFC* Medial femoral condyle

*LFC* Lateral femoral condyle

*MT* Medial tibial plateau

*LT* Lateral tibial plateau

*TR* Trochlea

*P* Patella

The ratios of the ICRS grade equality in 6 recipient sites are presented in Table 5. The ratios were determined to be between 72.7% and 89.3%. The ratios of the equality of operative indication in 6 recipient sites are presented in Table 6. The ratios were found to be between 90.0% and 96.0%. There were no significant differences in the sites of the both of the above ratios. In cartilage injuries, discrepant cases of operative indication were found in 50 recipient sites, and the surgical findings documented an area of injury of less than 1 cm diameter in 45 cases (90%). The recipient sites and areas of injury in another 5 cases were documented as follows: 15 × 20 mm in LFC (MRI grade 0, arthroscopy grade 4), 12 × 20 mm in MT (MRI grade 3, arthroscopy grade 2), 20 × 30 mm in TR (MRI grade 0, arthroscopy grade 3), 11 × 18 mm in TR (MRI grade 0, arthroscopy grade 4), and 15 × 20 mm in TR (MRI grade 2, arthroscopy grade 3). Our findings suggest that the diagnostic critical point of cartilage injuries was 1 cm in diameter, with injuries smaller than 1 cm being inconsistently detected on MRI.

**Table 5:** The ratio of the grade equality in 6 recipient sites

	MFC	LFC	MT	LT	TR	P
Grade Equality ratio	74.3%	89.3%	80.5%	84.7%	72.7%	74.7%

*MFC* Medial femoral condyle

*LFC* Lateral femoral condyle

*MT* Medial tibial plateau

*LT* Lateral tibial plateau

*TR* Trochlea

*P* Patella

**Table 6:** The ratio of the equality of operative indication in 6 recipient sites

	MFC	LFC	MT	LT	TR	P
Operative indication equality ratio	93.9%	93.3%	96.0%	97.3%	90.0%	95.3%

*MFC* Medial femoral condyle

*LFC* Lateral femoral condyle

*MT* Medial tibial plateau

*LT* Lateral tibial plateau

*TR* Trochlea

*P* Patella

**Fig. 2:**
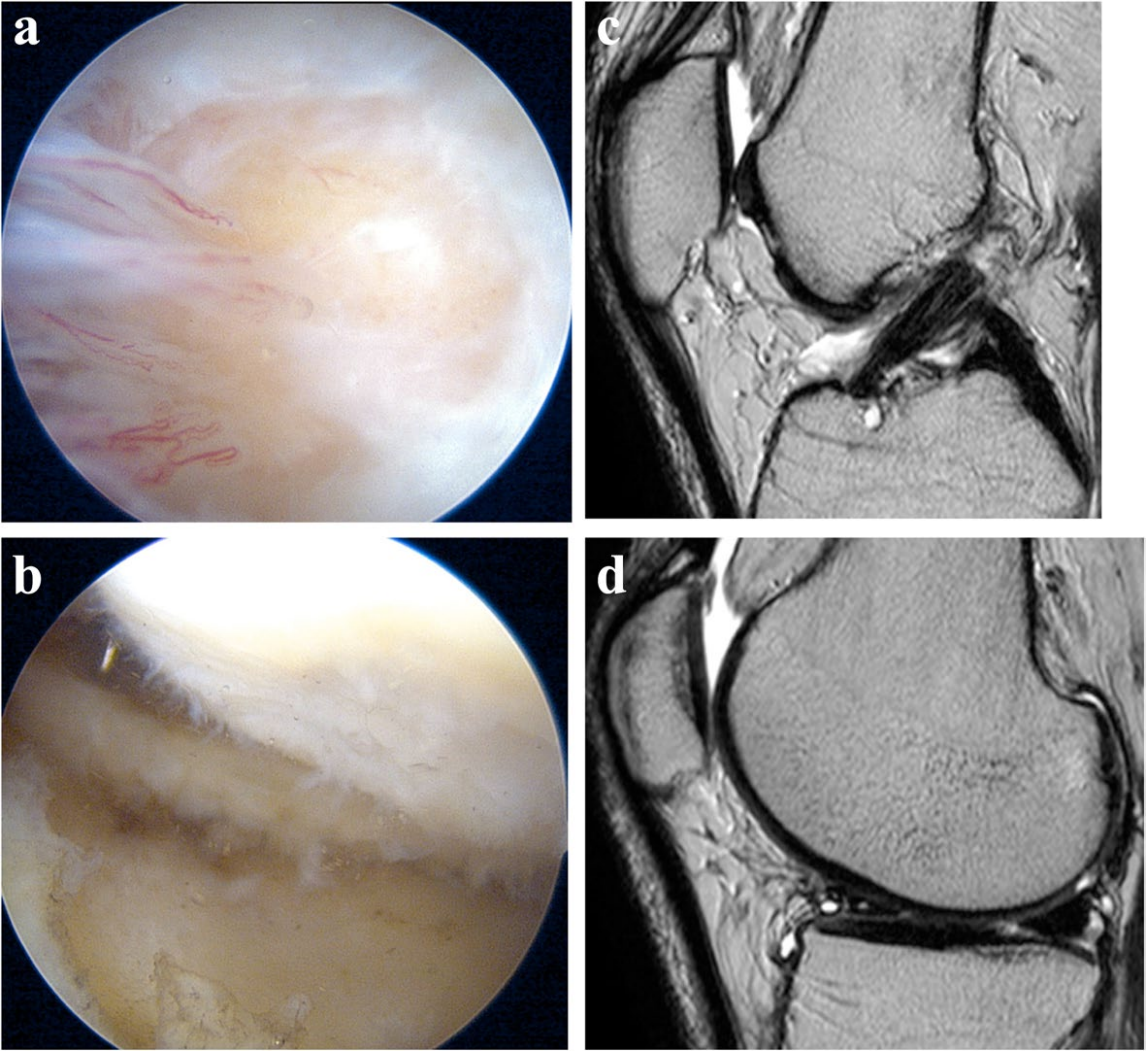
The oversight in the preoperative MRI case. **a** The arthroscopy showed 11 × 18 mm grade 4 cartilage injury in the trochlea. **b** The arthroscopy showed 10 × 10 mm grade 3 cartilage injury in the lateral femoral condyle. **c** His preoperative MRI showed the unevenness of articular cartilage with associated effusions in T2-weighted sequences in the trochlea. **d** His preoperative MRI showed the unevenness of articular cartilage with associated effusions in T2-weighted sequences in the lateral femoral condyle

The preoperative oversight case was shown in Fig. 2. This patient was 61 years-old, and was experiencing left knee pain with a catching sensation. We diagnosed a lateral meniscal tear and suspected the cartilage would appear to be normal. His arthroscopy revealed an 11 × 18 mm grade 4 trochlear cartilage injury (Fig. 2a), and a 10 × 10 mm grade 3 cartilage injury on the LFC (Fig. 2b). Retrospective review of his preoperative MRI showed unevenness of the articular cartilage with effusions in T2-weighted sequences in TR (Fig. 2c) and LFC (Fig. 2d). These changes were not read by the original doctor and are considered to be a reading oversight.

## Discussion

The summary of this study was in the following: 147 cases and 150 knee joints were enrolled in this study. We compared their preoperative MRI to the surgical findings of their knee arthroscopy. Preoperative MRIs found to have unevenness of articular cartilage along with the finding of inflammatory edema and effusions in T2-weighted sequences or irregularity of the subchondral bone in T1-weighted sequences (Fig. 1) were documented to have articular cartilage injuries. The sensitivity of MRI detection of ACL injuries was significantly higher than that in cartilage injuries (*P* = 0.0083), and the sensitivity in injuries of the MM was significantly higher than those in cartilage injuries of MT (*P *= 0.0465) and P (*P* = 0.0233). Regarding cartilage injuries, the sensitivity in MFC (*P* = 0.0697) and LT (*P* = 0.0640) were consistently higher than that in P. The ratios of the ICRS grade equality in 6 recipient sites were determined to be between 72.7% and 89.3%. The ratios of the equality of operative indication in these same 6 recipient sites were measured between 90.0% and 96.0%. The critical detection point of cartilage injuries was found to be >  = to 1 cm with injuries < 1 cm being more likely to be undetected on MRI.

Articular cartilage defects of the knee are frequently observed in most patient population. Prospective survey of 993 consecutive arthroscopic knee surgeries found articular cartilage pathology in 66% and a localized cartilage defect in 20% of the patients. Most lesions were found to be isolated high-grade lesions located on the femur [1]. Hjelle reported on articular cartilage defects in 1,000 consecutive knee arthroscopies. Focal chondral or osteochondral defects were found in 19% of the patients. The main focal chondral or osteochondral defect was found MFC in 58%, patella in 11%, LT in 11%, LFC in 9%, trochlea in 6%, and MT in 5%. [18]. Jones reported the most frequent lesion location as being the MFC [20]. Souza also reported that the MFC had the highest incidence of cartilage defects, and a high incidence of MM lesions was observed in subjects without MFC lesions [33]. In our study, the articular cartilage pathology existed in 22% of patients, and the most frequent lesion detected involved the MFC, which is consistent with the previous studies.

Fritz reported MRI findings of cartilage derangements in the following [12]: early chondral degeneration, cracks or fissures, chondral defects, the geographic appearance of a loose body, and osteochondral lesions. Early chondral degeneration appears on MRI as low signal intensity or high signal intensity chondral tissue that has lost the normal stratified and layered appearance. Cracks or fissures are commonly seen in the surface of articular cartilage and may be well seen with MRI as well as with arthroscopy. Chondral defects are recognized on MRI as fluid extending into and replacing the articular cartilage. The diagnosis of a chondral defect seen on MRI should prompt a search for associated loose bodies. Osteochondral lesions in the knee can be detected and well characterized with MRI without the need for special pulse sequences. Chang insisted that MRI evaluated the thickness and congruity of the articular surface in suspected articular cartilage injuries [4]. Kijowski reported that higher grades of articular cartilage defects are frequently associated with a greater depth and cross-sectional area of subchondral bone marrow edema [21]. We diagnosed articular cartilage injuries in MRI exhibiting unevenness of articular cartilage and associated inflammatory edema or effusions in T2-weighted sequences or in those exhibiting irregularity of subchondral bone in T1-weighted sequences. Other studies have reported on the diagnosis of cartilage degeneration with MRI. T1 rho and T2 MRI are complementary and reproducible methods for quantitatively and noninvasively monitoring regeneration [19]. T2 values increase with the increasing grade of cartilage damage and have a statistically significant positive correlation with ICRS scores [32].

Comparing the sensitivity of the recipient sites to preoperative MRI findings in diagnosing cartilage injuries, Danieli reported that the sensitivity of MRI was 76.4% (patella), 88.2% (trochlea), 69.7% (MFC), 85.7% (MT), 81.8% (LFC) and 75% (lateral plateau) [6]. Cartilage injuries affecting the MFC or the medial patellar facet were frequently missed by MRI [38]. Svard reported that modulus and T2 weighted images showed significant topographical variation. In the anterior medial condyle the modulus showed a negative association with the presence of an injury [36]. In an ACL-reconstructed knee, T2 values of the cartilage of the central aspect of the MFC at the 2-year follow-up were significantly elevated compared with native control knees [35]. In our study, the sensitivity, specificity and accuracy regarding injuries located on the MFC were higher than for injuries at other sites.

MRI appears to be less accurate than arthroscopy in diagnosing low-grade lesions particularly femorotibial lesions but is nearly equivalent to arthroscopy for high-grade lesions [10, 24]. T2 mapping can be useful for detecting moderate or severe cartilage damage, and the apparent diffusion coefficient can be used to detect early stage cartilage damage [37]. For grade III and IV lesions, 3-T MRI combined with three-dimensional double-echo steady-state cartilage-specific sequences represents an accurate diagnostic tool. For grade II lesions, the technique demonstrates moderate sensitivity, while for grade I lesions, the sensitivity is quite low [23]. MRI accuracy correlated negatively with patient age for articular cartilage damage when compared to arthroscopic findings [22]. MRI underestimated the defect area by an average of 70% compared with arthroscopic visualization [3]. No prior reports have discussed or determined the diagnostic threshold of MRIs for detecting injuries. The ratios for the equality of surgical indications based on surgery versus MRI were calculated to be between 90.0% and 96.0%, when unevenness of articular cartilage with concurrent inflammatory edema or effusions and the irregularity of subchondral bone are considered. The MRI threshold for detecting injuries consistently was 1 cm in longest diameter.

The sensitivity and specificity of MRI were found to be 87% and 93%, respectively, for ACL tears; 89% and 88%, for MM tears, and 78% and 95%, for lateral meniscus tears [29]. In a pediatric adolescent patient, the sensitivity and the specificity of 3 T MRI were 81% and 90.9% for MM injuries, 68.8% and 93% for lateral meniscus injuries, and 97.9% and 98.6% for ACL injuries, respectively [31]. Eijgenraam reported that the strongest correlation between MRI findings and radiographic OA was found in the medial femoral cartilage and the weakest correlation was found in the anterior horn of the MM [9]. Koch insisted that the highest accuracy was observed in MM and in ACL findings [22]. Porter reported that MRI was less accurate than clinical assessment for the diagnosis of lateral meniscal tears [30]. In our study, the sensitivity in MRI detection of ACL injuries was significantly higher than that in MRI detection of any cartilage injuries in any location of the cartilage, and the sensitivity in MRI detection of injuries in the MM was significantly higher than those in the cartilage injuries of MT and P.

One limitation of our study was the modest number of cases (147 cases and 150 knees). A second limitation is that this is a single institution study and a future multi-institutional study is warranted.

In conclusion, the diagnostic sensitivity of MRI in the detection of cartilage injuries was significantly lower than the sensitivity in the detection of ACL and meniscal injuries. The ratios of the equality of operative indication was determined to be between 90.0% and 96.0%, if we consider the unevenness of articular cartilage or the irregularity of subchondral bone. The diagnostic threshold for MRI was 1 cm in longest dimension which makes it effective as a diagnostic tool in clinical practice.

## Supplementary Information

**Additional file 1.**

## Data Availability

Our findings suggest that the diagnostic critical point of cartilage injuries was 1 cm in diameter, with injuries smaller than 1 cm being inconsistently detected on MRI.
