# Supplementary material for: Preoperative diagnosis of knee cartilage, meniscal, and ligament injuries by magnetic resonance imaging
Source: J Exp Orthop. 2023 Apr 20;10:47. doi: 10.1186/s40634-023-00595-y (PMC10119346; doi:10.1186/s40634-023-00595-y)
Supplement: Supplementary file 1 — Additional file 1. [file 40634_2023_595_MOESM1_ESM.pdf]

審 査 結 果 通 知 書

平成２５年４月１５日

申　請　者　　中川　泰彰　　殿

京都医療センター倫理委員会
委員長　　塚原　徹也

受付番号　　１３－２０

申請課題名　　自家骨軟骨移植術の術後成績調査に関する後向き研究

上記の申請課題につき、平成２５年４月１５日開催の倫理委員会での審査結果を下記のとおり通知します。

判　定	承　　認
理　由<br>・<br>その他	
